# Periostin expression and its supposed roles in benign and malignant thyroid nodules: an immunohistochemical study of 105 cases

**DOI:** 10.1186/s13000-021-01146-8

**Published:** 2021-09-25

**Authors:** Kimihide Kusafuka, Masaru Yamashita, Tomohiro Iwasaki, Chinatsu Tsuchiya, Aki Kubota, Kazuki Hirata, Akinori Murakami, Aya Muramatsu, Kazumori Arai, Makoto Suzuki

**Affiliations:** 1 Department of Pathology, Shizuoka General Hospital, Shizuoka, Japan; 2 Department of Otorhinolaryngology-Head and Neck Surger, Kagoshima University, Kagoshima, Japan

## Abstract

**Background:**

Thyroid tumors are often difficult to histopathologically diagnose, particularly follicular adenoma (FA) and follicular carcinoma (FC). Papillary carcinoma (PAC) has several histological subtypes. Periostin (PON), which is a non-collagenous extracellular matrix molecule, has been implicated in tumor invasiveness. We herein aimed to elucidate the expression status and localization of PON in thyroid tumors.

**Method:**

We collected 105 cases of thyroid nodules, which included cases of adenomatous goiter, FA, microcarcinoma (MIC), PAC, FC, poorly differentiated carcinoma (PDCa), and undifferentiated carcinoma (UCa), and immunohistochemically examined the PON expression patterns of these lesions.

**Results:**

Stromal PON deposition was detected in PAC and MIC, particularly in the solid/sclerosing subtype, whereas FA and FC showed weak deposition on the fibrous capsule. However, the invasive and/or extracapsular regions of microinvasive FC showed quite strong PON expression. Except for it, we could not find any significant histopathological differences between FA and FC. There were no other significant histopathological differences between FA and FC. Although PDCa showed a similar PON expression pattern to PAC, UCa exhibited stromal PON deposition in its invasive portions and cytoplasmic expression in its carcinoma cells. Although there was only one case of UCa, it showed strong PON immunopositivity. PAC and MIC showed similar patterns of stromal PON deposition, particularly at the invasive front.

**Conclusions:**

PON may play a role in the invasion of thyroid carcinomas, particularly PAC and UCa, whereas it may act as a barrier to the growth of tumor cells in FA and minimally invasive FC.

**Supplementary Information:**

The online version contains supplementary material available at 10.1186/s13000-021-01146-8.

## Introduction

Papillary carcinoma (PAC) is the most common tumor in the thyroid gland [1]. Among follicular thyroid lesions, difficulties are associated with differentiating between follicular adenoma (FA) and follicular carcinoma (FC) [2]. Since FA and FC, particularly the microinvasive type, exhibit similar cellular atypia, the key to their diagnosis is based on the presence/absence of capsular invasion.

Tumors are composed of parenchymal tissue (the tumor cells themselves) and the stroma, which contains the extracellular matrix (ECM) and myofibroblasts (cancer-associated fibroblasts: CAF). The stroma is not only a supportive tissue, it also regulates the migration, differentiation, and/or growth of tumor cells in the tumor microenvironment [3]. Periostin (PON) is a non-collagenous ECM molecule that is deposited on the periosteum and periodontal ligaments [4, 5]. PON was recently shown to be expressed in several cancers, including oral and breast cancers, and plays direct roles in the invasion and migration of tumor cells [6–9]. Moreover, PON-positive cancers have poor prognoses. We herein examined the expression and localization of PON in thyroid nodules.

## Materials and methods

We randomly selected 105 cases of thyroid nodules from our institution’s pathology files (study period: 2014–2018). They included 10 cases of adenomatous goiter (AG), 13 cases of microcarcinoma (MIC), 20 cases of FA, 18 cases of FC, 41 cases of PAC, 2 cases of poorly differentiated carcinoma (PDCa), and one case of undifferentiated carcinoma (UCa). As negative controls, background normal thyroid tissues were selected in matching cases. The clinicopathological features of the cases used in the present study were summarized in Supplemental Table 1.

We re-diagnosed each case based on hematoxylin and eosin (H&E) staining and selected specimens that exhibited typical histology. Immunohistochemical staining was performed on 4-μm-thick sections, which were cut from formalin-fixed and paraffin-embedded tissue. Immunostaining was performed according to the relevant manufacturer’s instructions using a Leica BOND MAX automated immunostainer (Leica, Bannockburn, IL, USA) or Ventana Benchmark GX automatic immunostainer (Roche Tissue Diagnosis, Oro Valley, AZ, USA). The antibodies used in the present study are summarized in Table 1. Tumors were considered to be diffusely positive (++), positive (+), focally positive (F+), and negative when ≥50, 10–49, 1–9, and 0% of neoplastic cells were positive, respectively. In the immunohistochemical assessment of PON expression, stromal localization was recorded as S and cytoplasm localization as CY. We used ImageJ (National Institutes of Health, Bethesda, MD, USA) to estimate the percentage of Ki-67-positive tumor cells.

**Table 1:** Antibodies used in the present study

Antigen	Clone	Source	Equipment	Antigen retrieval
PON	(P)	Abcam PLC (Cambridge, UK)	L	ER1 (30 min)
Galectin-3	9C4	Leica Biosystems (Bannockburn, IL, USA)	L	ER1 (30 min)
CK19	ROL108	DakoCytomation (Carpinteria, CA, USA)	L	ER2 (30 min)
HBME-1	HBME-1	DakoCytomation (Carpinteria, CA, USA)	L	ER2 (30 min)
Cyclin D1	SP4	Invitrogen (Carlsbad, CA, USA)	L	ER2 (20 min)
Ki-67	MIB1	DakoCytomation (Carpinteria, CA, USA)	R	CC1 (64 min)

*PON* Periostin, *CK* Cytokeratin, *HBME-1* Hector Battifora mesothelial-1, *(P)* Polyclonal antibody, *L* Leica BOND-MAX automatic immunostainer, *R* Roche VENTANA BenchMark ULTRA automatic immunostainer, *ER1* pH 6.0 (Leica Biosystems, Bannockburn, IL, USA), *ER2* pH 9.0 (Leica Biosystems, Bannockburn, IL, USA), *CC1* pH 8.5 (Roche Tissue Diagnostics, Basel, Switzerland)

## Results

### Histological findings

**Fig. 1:**
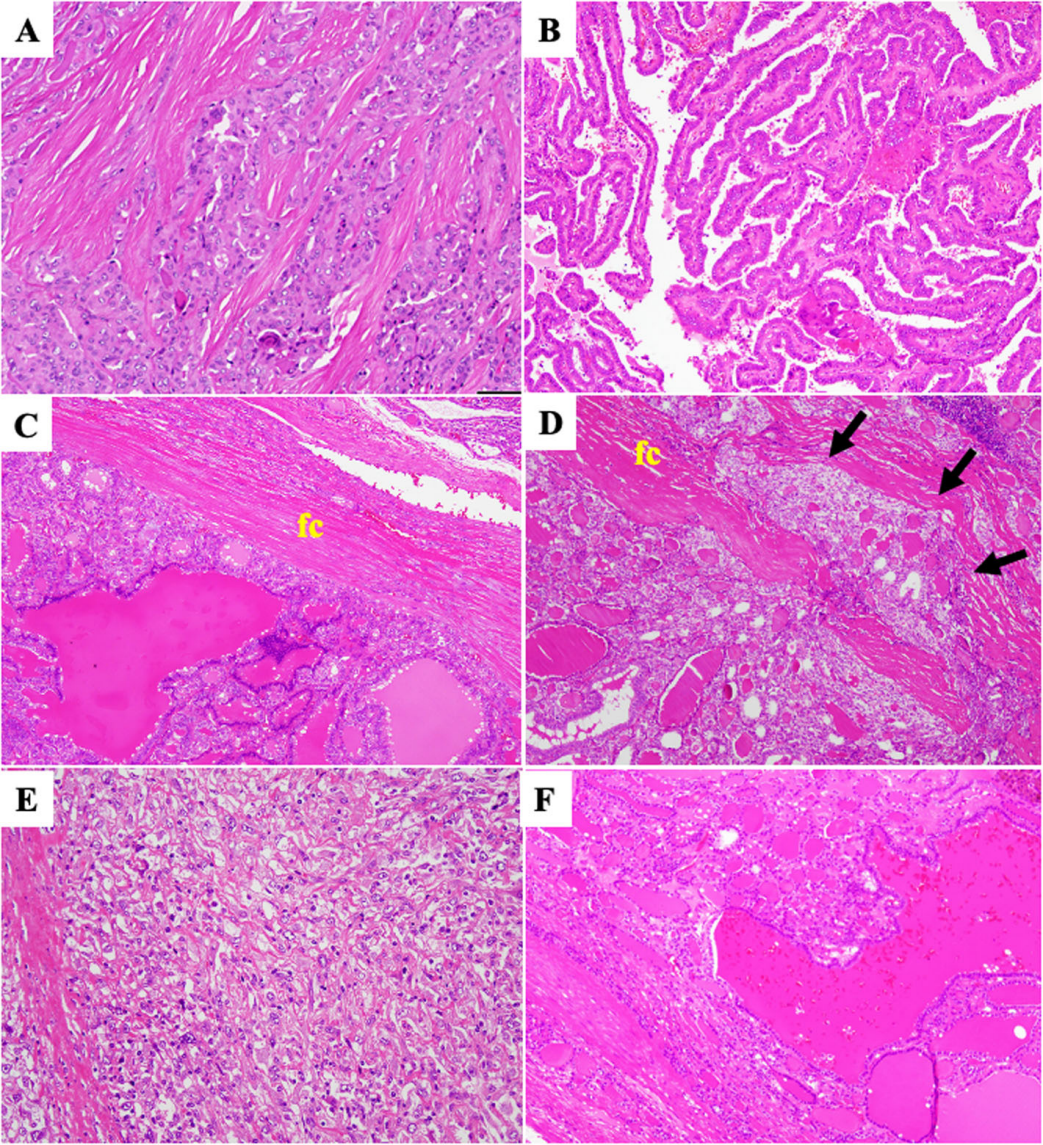
Histology of thyroid nodules. **A** Microcarcinoma (H&E). The nuclei of tumor cells showed a ground glass-like appearance, and tumor cells were surrounded by a sclerotic stroma. **B** Papillary carcinoma (H&E). Tumor cells, whose nuclei exhibited a ground glass-like appearance, formed irregular papillary structures. **C** Follicular adenoma (H&E). Follicles composed of tumor cells that exhibited mild nuclear atypia, but not capsular invasion, were observed (fc, fibrous capsule). **D** Minimally invasive follicular carcinoma (H&E). Tumor cells that exhibited mild atypia invaded the fibrous capsule (fc, fibrous capsule; arrows, capsular invasion). **E** Undifferentiated carcinoma (H&E). Polymorphous atypical short spindle-shaped cells that exhibited loose cell-cell adherence were arranged in fascicular structures or diffusely distributed. **F** Adenomatous goiter (H&E). Hyperplastic follicular cells without atypia were detected. Follicles contained colloid

All MIC were histologically classified as tiny PAC (Fig. 1A). There were 0 cases of the encapsulated (cap)/sclerosing (scl) subtype, 5 of the non-cap/scl subtype, 6 of the cap/non-scl subtype, and 2 of the non-cap/non-scl subtype. All PAC belonged to the well-differentiated type (Fig. 1B), and, according to their macroscopic findings, there were 23 cases of the localized/solid subtype, 7 of the localized/cystic subtype, and 11 of the mixed subtype. The present study did not include any cases of the diffuse scl subtype of PAC. FA showed dense follicle proliferation and thick fibrous capsules without invasion (Fig. 1C). On the other hand, 15 out of the 18 cases of FC harbored dense fibrous capsules with extracapsular invasion, which were subclassified into minimally invasive FC (MinI-FC) (Fig. 1D). The remaining 3 cases of FC did not possess fibrous capsules, and were classified as widely invasive FC (WI-FC). PDCa showed a solid and/or insular pattern. UCa was composed of densely packed proliferating spindle-shaped cells and polygonal cells, which exhibited marked cellular atypia, and a small focus of the follicular variant of PAC (Fig. 1E). AG did not possess fibrous capsules, but was well-defined. Some areas exhibited dense follicle proliferation, whereas others showed loosely distributed follicles with an edematous stroma (Fig. 1F).

### Immunohistochemical analysis of PON expression

The immunohistochemical results for PON are summarized in Table 2.

**Table 2:** Immunohistochemical results for PON

	MC		PAC		FC		PDCa		UCa		AG		FA	
PON	S	CY	S	CY	S	CY	S	CY	S	CY	S	CY	S	CY
(++)	38%	0%	54%	0%	0%	0%	0%	0%	100%	0%	0%	0%	0%	0%
(+)	31%	0%	24%	0%	0%	0%	0%	0%	0%	100%	0%	0%	0%	0%
(F+)	15%	0%	15%	0%	28%	0%	100%	50%	0%	0%	10%	0%	17%	0%
(−)	15%	100%	7%	100%	72%	100%	0%	50%	0%	0%	90%	100%	83%	100%
fc	38%	N.A.	27%	N.A.	72%	N.A.	N.A.	N.A.	N.A.	N.A.	(20%)	N.A.	89%	N.A.

*PON Periostin, *MIC* Microcarcinoma, *PAC* Papillary thyroidal carcinoma, *FC* Follicular carcinoma, *PDCa* Poorly differentiated carcinoma, *UCa* Undifferentiated carcinoma, *AG* Adenomatous goiter, *FA* Follicular adenoma

*S Stroma, *CY* Cytoplasm, *fc* Fibrous capsule, (−) Negative [0%], *(F+)* Focally positive [1–9%], (+) Positive [10–49%], (++) Diffusely positive [≥50%], *L.I*. Labeling index, *N.A.* Not available

**Fig. 2:**
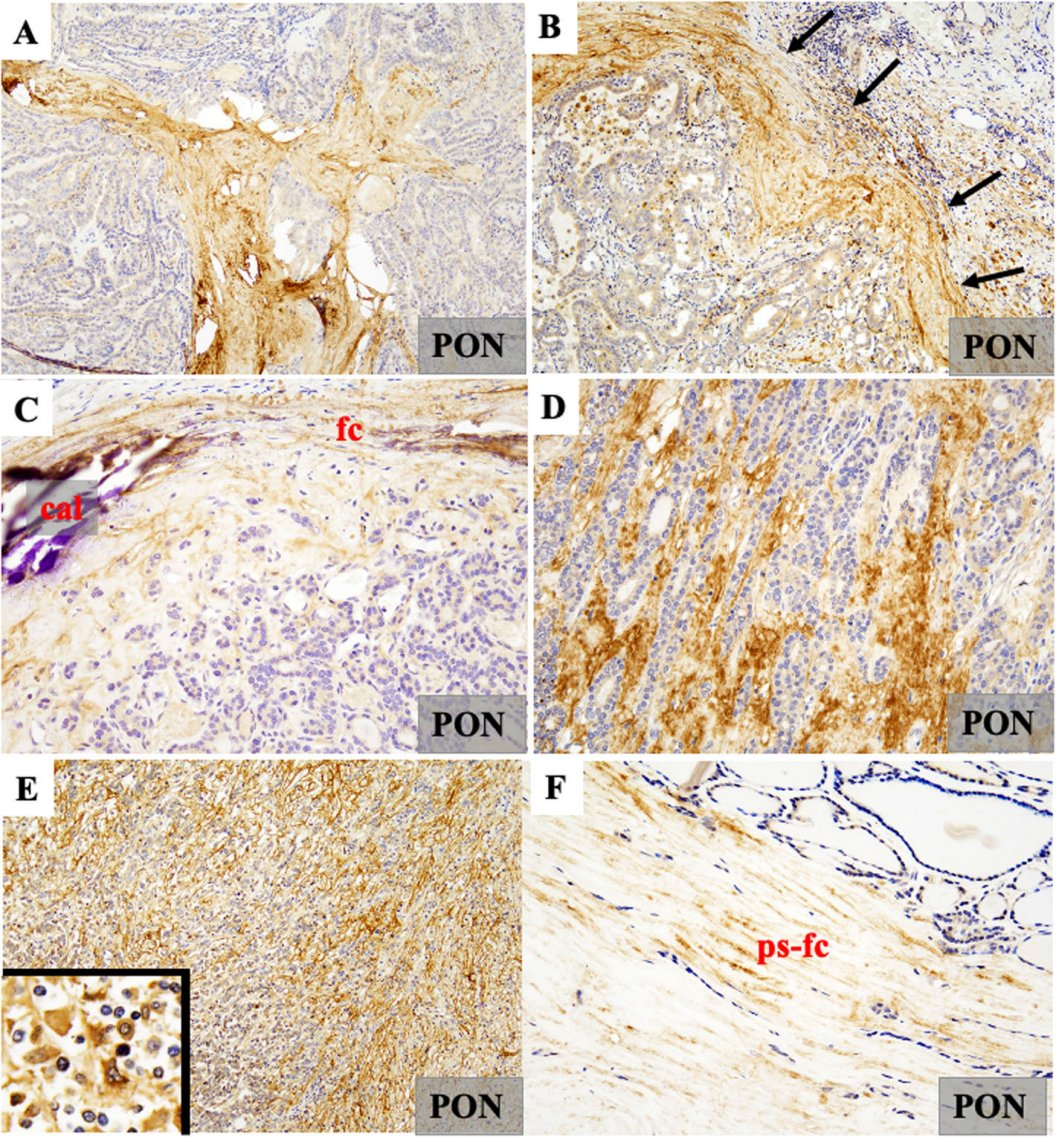
Results of immunohistochemical staining for periostin (PON) in each thyroid nodule. **A** Microcarcinoma. Immunopositivity for PON was observed in the sclerotic stroma. **B** Papillary carcinoma. Moderate to strong immunopositivity for PON was observed in the sclerotic stroma around the neoplastic follicles. **C** Follicular adenoma. Weak immunopositivity for PON was noted in the fibrous capsule (fc), together with some calcification (cal). **D** Widely invasive follicular carcinoma. Diffuse immunopositivity for PON was detected in the sclerotic stroma in the invasive regions. **E** Undifferentiated carcinoma. Strong immunopositivity for PON was diffusely observed in the stroma around cancer cells. (E, inset) PON immunoreactivity was also observed in the cytoplasm of cancer cells. **F** Adenomatous goiter. Very weak signals for PON were found in the pseudo-capsule (ps-fc) around the nodule in adenomatous goiter

The scl subtype of MIC showed moderate positivity for PON, whereas the non-scl subtype did not express PON. In the cap and non-cap subtypes of MIC, PON localized to the fibrous capsule-like stroma and hyalinous stroma, except in non-scl-type MIC (Fig. 2A).

The localized cystic subtype of PAC showed weak positivity for PON on cyst walls, whereas moderate and strong positivities for PON were observed in the hyaline stroma in the other subtypes of PAC. However, immunopositivity for PON was not observed in the cytoplasm of cancer cells (Fig. 2B). PON localized around calcified areas or psammoma bodies. The follicular variant of PAC showed scant stromal tissue and weak positivity for PON (Supplemental data 1). PAC cases that exhibited direct invasion into the thyroid capsule exhibited stronger immunopositivity for PON than the other cases.

FA showed weak positivity for PON on the fibrous capsule (Fig. 2C), which was similar to that observed in MinI-FC. However, WI-FC showed stronger positivity for PON at the invasive tip than in the inner stroma (Fig. 2D).

Since PDCa exhibited medullary growth, PON localized to the scant stroma. UCa showed both stromal and cytoplasmic positivity for PON (Fig. 2E). AG showed very weak or no positivity for PON (Fig. 2F).

### Immunohistochemical analysis of other markers

The immunohistochemical results obtained for other markers are summarized in Table 3.

**Table 3:** Immunohistochemical results for other markers

	MIC	PAC	FC	PDCa	UCa	AG	FA
Gal-3							
(++)	100%	73%	0%	0%	0%	0%	0%
(+)	0%	27%	0%	0%	100%	0%	0%
(F+)	0%	0%	11%	50%	0%	0%	28%
(−)	0%	0%	73%	50%	0%	100%	72%
CK19							
(++)	75%	88%	0%	0%	0%	0%	0%
(+)	25%	61%	0%	0%	100%	0%	0%
(F+)	0%	0%	33%	50%	0%	20%	28%
(−)	0%	0%	67%	50%	0%	80%	72%
HBME-1							
(++)	75%	85%	0%	0%	0%	0%	0%
(+)	17%	10%	0%	0%	0%	0%	0%
(F+)	8%	0%	11%	50%	0%	10%	11%
(−)	0%	5%	89%	50%	100%	90%	56%
Cyclin D1							
(++)	100%	95%	0%	0%	0%	0%	0%
(+)	0%	0%	0%	50%	0%	0%	0%
(F+)	0%	0%	11%	50%	100%	0%	17%
(−)	0%	5%	89%	0%	0%	100%	83%
Ki-67 L.I. (mean percentage							
	5.2%	7.1%	3.3%	10%	65%	< 1%	2.9%

*Gal-3 Galectin-3, *HBME-1* Hector Battifora mesothelial-1, *CK* cytokeratin

*MIC Microcarcinoma, *PAC* Papillary thyroidal carcinoma, *FC* Follicular carcinoma, *PDCa* Poorly differentiated carcinoma, *UCa* Undifferentiated carcinoma, *AG* Adenomatous goiter, *FA* Follicular adenoma

*(−), negative [0%]; (F+), focally positive [1–9%]; (+), positive [10–49%]; (++), diffusely positive [≥50%]; L.I., labeling index

**Fig. 3:**
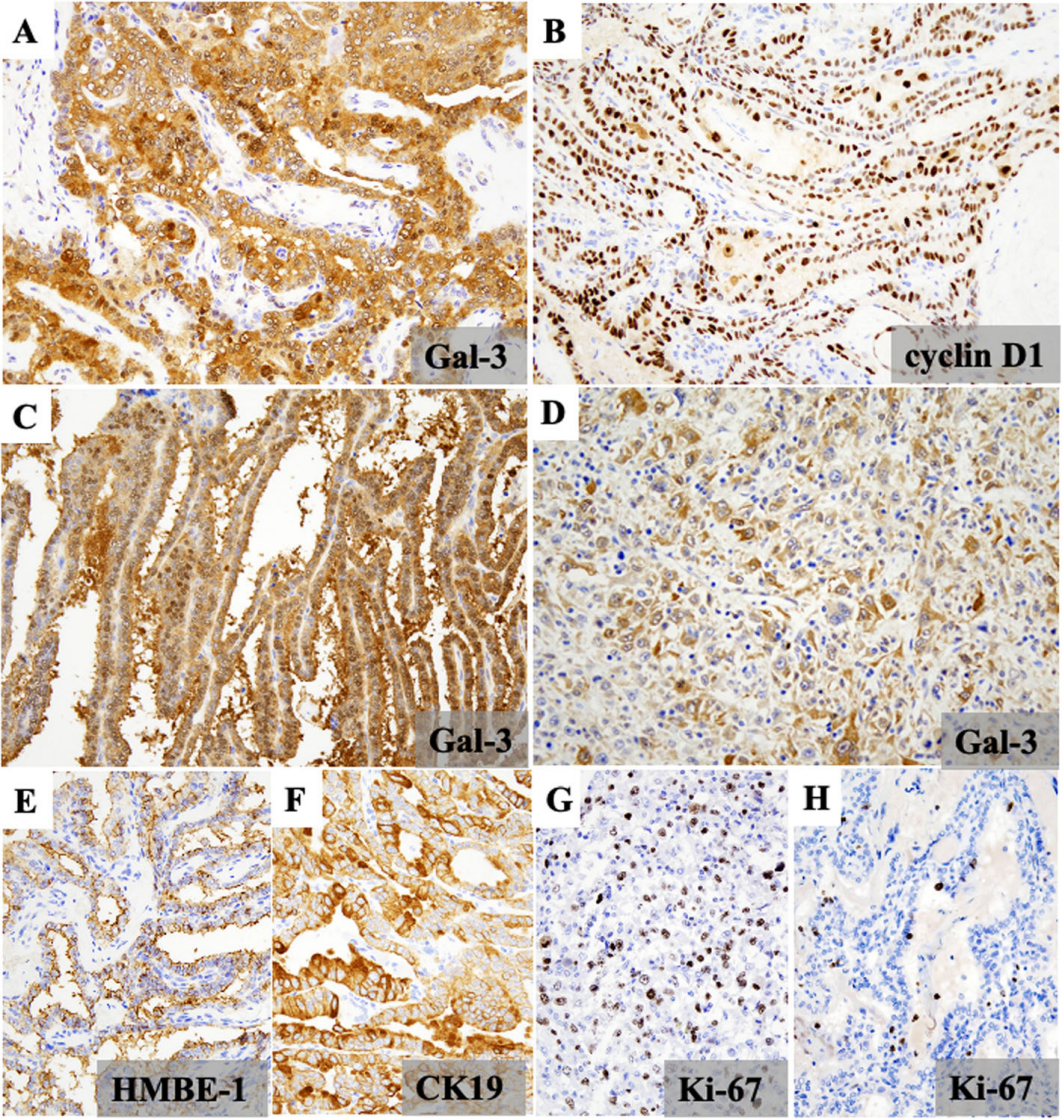
Immunohistochemical staining results for other thyroid tumor markers. In microcarcinoma, cancer cells exhibited strong positivity for galectin-3 (Gal-3) (**A**) and nuclear positivity for cyclin D1 (**B**). In papillary carcinoma, the cytoplasm of cancer cells was diffusely positive for Gal-3 (**C**). Undifferentiated carcinoma showed cytoplasmic immunopositivity for Gal-3 with a mosaic-like pattern (**D**). In papillary carcinoma, the luminal side of papillary structures was positive for Hector Battifora mesothelial 1 (HBME-1) (**E**), and tumor cells were diffusely positive for cytokeratin 19 (**F**). In undifferentiated carcinoma, cancer cells were diffusely positive for Ki-67 (**G**), whereas tumor cells in papillary carcinoma were sporadically positive for Ki-67 (**H**)

PAC and MIC were mainly positive for cytokeratin (CK) 19, whereas AG, FA, and FC were only focally immunopositive for CK19. MIC, PAC, and WI-FC were also positive for galectin-3 (Gal-3) (Fig. 3A and C), whereas AG and FA were immunonegative for Gal-3. MinI-FC showed focal immunopositivity for Gal-3. MIC and PAC exhibited diffuse nuclear immunopositivity for cyclin D1 (Fig. 3B), whereas FA and AG were negative for cyclin D1. FC, particularly the WI type; PDCa; and UCa were partially immunopositive for Hector Battifora mesothelial 1 (HBME-1) and cyclin D1. UCa showed mosaic patterns of immunopositivity for Gal-3(Fig. 3D) and Ki-67 (Fig. 3G). PAC and MIC were immunopositive, to varying extents, for HBME-1 (Fig. 3E) and CK19 (Fig. 3F), whereas FA and FC were negative or only focally positive for HBME-1. AG was immunonegative for HBME-1. FC, particularly the WI type; PDCa; and UCa was partially immunopositive for HBME-1 and cyclin D1.

The Ki-67 L.I. of AG, MIC, PAC, FA, FC, PDCa, and UCa were < 1, 1–5, 2–9, 3, 3, 11, and 65%, respectively (Fig. 3G and H). In pT4a and/or pEx1 (thyroid capsular invasion+) PAC, the Ki-67 L.I. was slightly higher. The Ki-67 L.I. of FA and FC were similar. UCa had a significantly higher Ki-67 L.I. than the other thyroid tumors/nodules.

## Discussion

PON is a glycoprotein that consists of a 93.3-kDa homodimer and was initially described as osteoblast-specific factor 2 in 1993 [4, 10]. Although PON was previously considered to contribute to the adhesion of pre-osteoblasts [4], it has since been shown to play important roles in the formation of collagen fibers, cell-cell adhesion, and wound repair [11–14]. Moreover, PON has been associated with invasion capacity and stromal formation in several cancer types [6–9, 15, 16]. CAF secrete epidermal growth factor and insulin-like growth factor-1, which promote tumor growth. Moreover, CAF secrete hepatocyte growth factor/scattering factor, which promote cell migration [3]. On the other hand, CAF are also involved in the construction and/or remodeling of the ECM; i.e., they play a role in dissolving type I, III, and V collagen, fibronectin, and laminin, and, thus, promote the migration and invasion of cancer cells [17]. PON has been implicated in non-small cell lung cancer, oral cancer, and liver cancer [6–9, 15, 16]. In numerous cancer types, cancer cells themselves have been reported to secrete PON; however, stromal CAF also secrete PON, which promotes the migration and/or invasion of cancer cells [18, 19]. Ratajczak-Wielgomas et al. showed that benign lesions of the breast showed no stromal PON deposition, whereas PON was deposited around malignant mammary ducts in ductal carcinoma in situ, and was more widely deposited in the cancer stroma of invasive ductal carcinoma of the breast, particularly in cases showing high-grade histological atypia [19]. PON secreted by CAF is not only involved in tumor cell migration and invasion, but is also associated with tumor cell differentiation and high-grade malignancy via epithelial-mesenchymal transition [19, 20].

In the present study, we examined the expression and localization of PON in thyroid tumors. Cytoplasmic PON localization was not observed in our series, except in UCa. This may have been because the antibody used in our study recognized a different epitope to those used in previous studies. Alternatively, it may have been due to the slow growth of thyroid tumors (even malignant tumors), except for UCa.

Since AG is not associated with CAF recruitment and is a hyperplastic lesion, AG did not show any PON expression. On the other hand, PON expression was low on fibrous (pseudo-)capsules in tumors with fibrous capsules (i.e., cap-MIC, FA, and the localized/cystic type of PAC), whereas it was clearly expressed in the hyaline stroma of stroma-rich tumors. UCa, which is a rapidly growing tumor, showed weak positivity for PON in the stroma and cytoplasm of tumor cells. Based on these results, PON does not contribute to the growth capacity of thyroid tumors.

Two reverse transcription-polymerase chain reaction (RT-PCR)-based studies examined PON expression in PAC [21, 22]. The findings obtained indicated that PON mRNA expression was higher in tumor tissues than in normal tissues. Bai et al. detected high PON mRNA expression in cases of PAC involving extracapsular invasion, and reported that PON expression in PAC was higher at the invasive front and was related to loss of polarity in cancer cells [21]. However, the localization of PON in PAC remains unknown. In the present study, PON was deposited in the fibrous stroma in PAC, and larger deposits were observed in cases involving invasion into the thyroid capsule. There are 5 wild-type isoforms of PON. Using RT-PCR and direct DNA sequencing, Bai et al. identified three new isoforms of PON mRNA (variations were observed on the C-terminal side) in PAC [22]; however, their clinicopathological significance remains unclear. In urothelial carcinoma, some cases expressed variant I (loss of exons XVII, XVIII, and XXI), whereas others expressed variant II (loss of exons XVII and XXI) [23], and variant I was associated with in vitro invasiveness and in vivo metastatic capacity. Similar relationships may exist in PAC.

In the present study, we examined thyroid tumors other than PAC, and PON deposition was detected on the thick fibrous capsules of FA and MinI-FC. However, MIC showed stronger PON deposition than FA and MinI-FC. These results suggest that PON plays a role in capsular formation and/or acts as barrier against tumor extension or invasion, whereas PON in MIC plays a role in invasiveness rather than barrier function.

We immunohistochemically examined the expression of HMBE-1 and Gal-3. Consistent with previous findings, most cases of PAC were positive for both markers, whereas approximately 50% of cases of FA and 70% cases of MinI-FC showed the partial expression of these PAC markers [24, 25]. Immunohistochemical examinations of both HMBE-1 and Gal-3 expression are considered to be useful for the differential diagnosis of thyroid tumors. The high expression of cyclin D1 in MIC (tiny PAC type) was recently reported to positively correlate with extracapsular invasion [26], and the present study showed that all cases of MIC belonged to the tiny PAC type. Twelve (92%) out of the 13 MIC cases and 38 (93%) out of the 41 PAC cases were positive for cyclin D1; however, cyclin D1 positivity was not related to extracapsular invasion or lymph node metastasis.

## Conclusions

In conclusion, the present results suggested that stromal PON deposition in PAC was related to stromal formation and invasion, whereas PON deposition on the fibrous capsules of FA or MinI-FC may function as a barrier against tumor extension. Although PON may play different roles in each histological type of thyroid tumors, the biological roles of PON in thyroidal tumors remain unknown.

## Supplementary Information

**Additional file 1: Supplemental table 1**. Clinicopathological characteristics of thyroid nodules examined in the present study.

**Additional file 2: Supplemental Figure 1**: Follicular variant of papillary carcinoma (A: H&E). Weak signals for PON were observed in the scant stroma (B).

## Data Availability

The datasets used and analyzed during the present study are available from the corresponding author upon reasonable request.

## Abbreviations

PAC: Papillary carcinoma
FC: Follicular carcinoma
FA: Follicular adenoma
ECM: Extracellular matrix
CAF: Cancer-associated fibroblasts
PON: Periostin
PDCa: Poorly differentiated carcinoma
UCa: Undifferentiated carcinoma
MIC: Microcarcinoma
H&E: Hematoxylin and eosin
cap: Encapsulated
scl: Sclerosing
AG: Adenomatous goiter
MinI-FC: Minimally invasive follicular carcinoma
WI-FC: Widely invasive FC
CK: Cytokeratin
HBME-1: Hector Battifora mesothelial-1
Gal-3: Galectin-3
L.I.: Labeling index
RT-PCR: Reverse transcription polymerase chain reaction

## References

[1] Rosai J, Albores Saavedra J, Asioki S, Bolach ZW, Bogdova T, Chen H. Papillary carcinoma. In: Lloyd RV, Osamura RY, Kloppel G, Rosai J, editors. WHO classification of Tumours of endocrine organs. 4th ed. Lyon: IARC Press. 2017:81–91.

[2] LiVolsi V, Abdulkader Nalib I, Baloch ZW, Bartolazzi A, Chan JKC, DeLellis RA. Follicular thyroidal carcinoma. In: Lloyd RV, Osamura RY, Kloppel G, Rosai J, editors. WHO classification of Tumours of endocrine organs. 4th ed. Lyon: IARC Press. 2017:92–5.

[3] Santi A, Kugeratski FG, Zanivan S (2018). Cancer associated fibroblasts: the architects of stroma remodeling. Proteomics.

[4] Horiuchi K, Amizuka N, Takeshita S, Takamatsu H, Katsuura M, Ozawa H (1999). Identification and characterization of a novel protein, periostin, with restricted expression to periosteum and periodontal ligament and increased expression by transforming growth factor beta. J Bone Miner Res.

[5] Kudo A (2019). Periostin in bone biology. Adv Exp Med Biol.

[6] Nuzzo PV, Rubagotti A, Zinoli L, Salvi S, Boccardo S, Boccardo F (2016). The prognostic value of stromal and epithelial periostin expression in human breast cancer: correlation with clinical pathological features and mortality outcome. BMC Cancer.

[7] Shao R, Bao B, Bai X, Blanchette C, Anderson RM, Dang T (2004). Acquired expression of periostin by human breast cancers promotes tumor angiogenesis through up-regulation of vascular endothelial growth factor receptor 2 expression. Mol Cell Biol.

[8] Siriwardena BS, Kudo Y, Ogawa I, Kitagawa M, Kitajima S, Hatano H (2006). Periostin is frequently overexpressed and enhances invasion and angiogenesis in oral cancer. Br J Cancer.

[9] Jang SY, Park SY, Lee HW, Choi Y-K, Park K-G, Yoon GS (2016). The combination of periostin overexpression and microvascular invasion in related to a poor prognosis for hepatocellular carcinoma. Gut Liver.

[10] Takeshita S, Kikuno R, Tezuka K, Amann E (1993). Osteoblast-specific factor 2: cloning of a putative bone adhesion protein with homology with insert protein fasciclin I. Biochem J.

[11] Nunomura S, Nanri Y, Okawa M, Arima K, Mitamura Y, Yoshihara T (2018). Constitutive overexpression of periostin delays wound healing in mouse skin. Wound Repair Regen.

[12] Cobo T, Viloria CG, Solares L, Fontanil T, Gonzalez-Chamorro E, De Carlos F (2016). Role of periostin in adhesion and migration of bone remodeling cells. PLoS One.

[13] Zhao S, Wu H, Xia W, Chen X, Zhu S, Zhang S (2014). Periostin expression is upregulated and associated with myocardial fibrosis in human failing hearts. J Cardiol.

[14] Braun N, Sen K, Alscher MD, Fritz P, Kimmel M, Morelle J (2013). Periostin: a matricellular protein involved in peritoneal injury during peritoneal dialysis. Perit Dial Int.

[15] Nuzzo PV, Buzzatti G, Ricci F, Rubagotti A, Argellati F, Zinoli L (2014). Periostin: a novel prognostic and therapeutic target for genitourinary cancer?. Clin Genitourin Cancer.

[16] Takanami I, Abiko T, Koizumi S (2008). Expression of periostin in patients with non-small-cell lung cancer: correlation with angiogenesis and lymphangiogenesis. Int J Biol Markers.

[17] Nissen NI, Karsdal M, Willumsen N (2019). Collagens and cancer associated fibroblasts in the reactive stroma and its relation to cancer biology. J Exp Clin Cancer Res.

[18] Oh HJ, Bae JM, Wen XY, Cho NY, Kim JH, Kang GH (2017). Overexpression of POSTN in tumor stroma is a poor prognostic indicator of colorectal cancer. J Pathol Trans Med.

[19] Ratajczak-Wielgomas K, Grzegrzolka J, Piotrowska A, Gomulkiewicz A, Witkiewicz W, Dziegial P (2016). Periostin expression in cancer-associated fibroblasts of invasive ductal breast carcinoma. Oncol Rep.

[20] Kikuchi Y, Kunita A, Iwata C, Komura D, Nishiyama T, Shimazu K (2014). The niche component periostin is produced by cancer-associated fibroblasts, supporting growth of gastric cancer through ERK activation. Am J Pathol.

[21] Bai Y, Kakudo K, Nakamura M, Ozaki T, Li Y, Liu Z (2009). Loss of cellular polarity/cohesiveness in the invasive front of papillary thyroid carcinoma and periostin expression. Cancer Lett.

[22] Bai Y, Nakamura M, Zhou G, Li Y, Liu Z, Ozaki T (2010). Novel isoforms of periostin expressed in the human thyroid. J Clin Med.

[23] Kim CJ, Isono T, Tambe Y, Chano T, Okabe H, Okada Y (2008). Role of alternative splicing of periostin in human bladder carcinogenesis. Int J Oncol.

[24] Saleh HA, Jin B, Barnwell J, Alzohaili O. Utility of immunohistochemical markers in differentiating benign from malignant follicular-derived thyroid nodules. Diagn Pathol. 2010;5(1). 10.1186/1746-1596-5-9. 10.1186/1746-1596-5-9 PMC2831001 20181018

[25] Prasad ML, Pellegata NS, Huang Y, Nagaraja HN, de la Chapelle A, Kloos RT (2005). Galectin-3, fibronectin-1, CITED-1, HBME-1 and cytokeratin-19 immunohistochemistry is useful for the differential diagnosis of the thyroid tumors. Mod Pathol.

[26] Juan C-K, Kang Y-G, Bae J-S, Lim D-J, Choi Y-J, Lee K-Y (2010). Unique patterns of tumor growth related with the risk of lymph node metastasis in papillary thyroid carcinoma. Mod Pathol.

