# Supplementary material for: Periostin expression and its supposed roles in benign and malignant thyroid nodules: an immunohistochemical study of 105 cases
Source: Diagn Pathol. 2021 Sep 25;16:86. doi: 10.1186/s13000-021-01146-8 (PMC8465710; doi:10.1186/s13000-021-01146-8)
Supplement: Supplementary file 1 — Additional file 1: Supplemental table 1. Clinicopathological characteristics of thyroid nodules examined in the present study. [file 13000_2021_1146_MOESM1_ESM.docx]

**Supplemental data**

Supplemental table 1. Clinicopathological characteristics of thyroid nodules examined in the present study

|  | MIC | PAC | FC | PDCa | UCa | AG | FA |
| --- | --- | --- | --- | --- | --- | --- | --- |
| Mean age | 55 y/o | 56 y/o | 55 y/o | 68 y/o | 72 y/o | 57 y/o | 49 y/o |
| Age range | 33-74y/o | 14-74 y/o | 20-78 y/o | 32-71 y/o | 72 y/o | 35-77 y/o | 26-76 y/o |
| M/F | 2:9 | 1:2 | 1:2 | 1:1 | 0:1 | 3:7 | 3:7 |
| Case No. | 13 | 41 | 18 | 2 | 1 | 10 | 20 |
| pT factor |  |  |  |  |  |  |  |
| pT1a | 7 | X | X | X | X | - | - |
| pT1b | X | 8 | 2 | 0 | X | - | - |
| pT2 | X | 0 | 8 | 0 | X | - | - |
| pT3 | 6 | 28 | 8 | 2 | X | - | - |
| pT4a | X | 3 | 0 | 0 | X | - | - |
| pT4b | X | 2 | 0 | 0 | 1 | - | - |
| pEX factor |  |  |  |  |  |  |  |
| pEX0 | 7 | 11 | 16 | 0 | 0 | - | - |
| pEX1 | 6 | 28 | 2 | 2 | 0 | - | - |
| pEX2 | 0 | 2 | 0 | 0 | 1 | - | - |
| pN factor |  |  |  |  |  |  |  |
| pNx | 3 | 1 | 7 | 1 | 0 | - | - |
| pN0 | 4 | 9 | 10 | 1 | 1 | - | - |
| pN1 | 6 | 31 | 1 | 0 | 0 | - | - |
| pStage |  |  |  |  |  |  |  |
| X | 0 | 1 | 5 | 0 | 0 | - | - |
| I | 5 | 12 | 9 | 0 | 0 | - | - |
| II | 5 | 19 | 3 | 2 | 0 | - | - |
| III | 3 | 7 | 1 | 0 | 0 | - | - |
| IV A | 0 | 2 | 0 | 0 | 0 | - | - |
| IV B | 0 | 0 | 0 | 0 | 0 | - | - |
| IV C | 0 | 0 | 0 | 0 | 1 | - | - |
| Invasion |  |  |  |  |  |  |  |
| MinI | X | X | 15 | X | X | - | - |
| WI | X | X | 3 | X | X | - | - |

| *MIC, microcarcinoma; PAC, papillary thyroid carcinoma; FC, follicular carcinoma;  PDCa, poorly differentiated carcinoma; UCa, undifferentiated carcinoma; AG,  adenomatous goiter; FA, follicular adenoma | |
| --- | --- |
| *MinI, minimally invasive; WI, widely invasive  *X, theoretically impossible to estimate; -, impossible to estimate since it is  a benign lesion.  **Supplemental figure legend.**  Supplemental Figure 1: Follicular variant of papillary carcinoma (A: H&E).  Weak signals for PON were observed in the scant stroma (B). |  |
