# Supplementary figures and images for: Periostin expression and its supposed roles in benign and malignant thyroid nodules: an immunohistochemical study of 105 cases
Source: Diagn Pathol. 2021 Sep 25;16:86. doi: 10.1186/s13000-021-01146-8 (PMC8465710; doi:10.1186/s13000-021-01146-8)

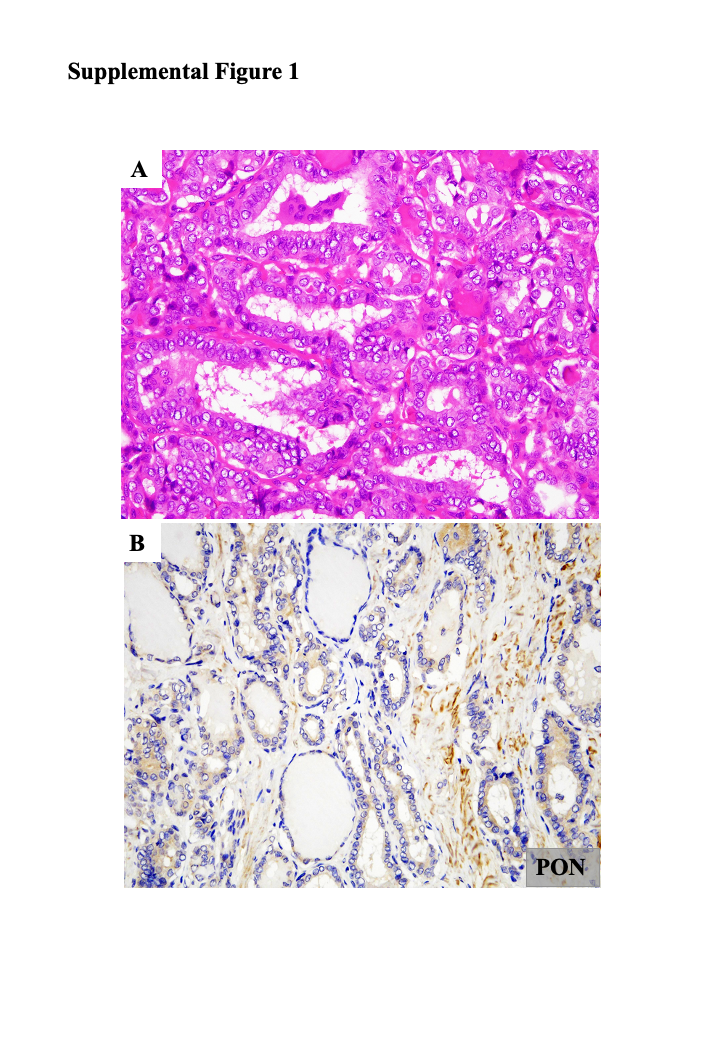

Supplement: Supplementary file 2 — Additional file 2: Supplemental Figure 1: Follicular variant of papillary carcinoma (A: H&E). Weak signals for PON were observed in the scant stroma (B). [file 13000_2021_1146_MOESM2_ESM.tiff]
